# The complete chloroplast genome of ornamental and medicinal *Callerya dielsiana* (Fabaceae)

**DOI:** 10.1080/23802359.2022.2105664

**Published:** 2022-08-01

**Authors:** Qi-Fei Yi, Li-Na Han, Ming-Chen Lin, Yong Tan, Lin Fu, Lei Duan, Hong-Feng Chen

**Affiliations:** a Guangdong Provincial Key Laboratory of Applied Botany, South China Botanical Garden, Chinese Academy of Sciences, Guangzhou, China; b College of Forestry and Landscape Architecture, South China Agricultural University, Guangzhou, China; c Guangdong Nanling National Nature Reserve, Shaoguan, China; d Zhongkai University of Agriculture and Engineering, Guangzhou, China

**Keywords:** *Callerya dielsiana*, chloroplast genome, tropical/subtropical liana

## Abstract

*Callerya dielsiana* is a Chinese endemic tropical/subtropical liana. We sequenced the complete chloroplast genome with the Illumina Hiseq X-Ten platform. The genome is obtained with 132,301 bp in length, lacking an inverted repeat (IR) region, contains 4 rRNAs, 30 tRNAs genes, and 76 protein-coding genes. The overall GC content is 33.9%. Based on the whole chloroplast genomes of 14 legume species, a phylogenetic tree is constructed and indicated that *C. dielsiana* belongs to the well-supported tribe Wisterieae. The tribe is sister to *Glycyrrhiza* and nested within the IRLC (Inverted Repeat-Lacking Clade) group of the subfamily Papilionoideae (Fabaceae).

The tropical/subtropical leguminous species *Callerya dielsiana* (Harms ex Diels) P.K.Lôc ex Wei and Pedley (2010) is an ornamental woody liana species endemic to southern China (Compton et al. 2019; Duan et al. 2021), which can also be used as medicinal plant to promote blood circulation and to dissipate blood stasis (Song et al. 1992; Gong 2010). Few study focused on genome of *C. dielsiana*, a better genomics knowledge of this species would benefit the future works on population genetics, diversity and gardening.

The fresh leaves of *C. dielsiana* was collected in Tianjing Mt., Guangdong Province, China (24°41′24″E, 113°3′36″N), and the voucher specimen was deposited at South China Botanical Garden, Chinese Academy of Science (IBSC; http://english.scbg.ac.cn/, Shi-Xiao Luo, luoshixiao@scbg.ac.cn) under the voucher number *L.Duan 2016022*. The total genomic DNA was extracted using CTAB approach (Doyle 1987), the cDNA library was prepared and sequenced with the Illumina Hiseq X-Ten platform (Illumina Inc., San Diego, CA). The sequences were filtered following the protocol of Yao et al. (2016), the resultant adaptor-free reads were then assembled with SPAdes 3.11 (Bankevich et al. 2012). The program of Dual Organellar GenoMe Annotator (DOGMA; Wyman et al. 2004) was used to annotate the assembly of complete chloroplast (cp) genome, which has been deposited in GenBank (accession number: MW007722). Raw reads were also deposited in the GenBank (SRA: SRR13871830; BioProject: PRJNA706880; Bio-Sample: SAMN18131232).

About 1.97 Gb raw reads of *C. dielsiana* were obtained. The cp genome, with 132,301 bp in length and lacking one inverted repeat (IR) region, contained 4 ribosomal RNA genes (rRNA), 30 transfer RNA genes (tRNA), and 76 protein-coding genes (CDS). Within the cp genome, we found 15 one-intron genes (*atpF*, *clpP*, *ndhA*, *ndhB*, *petB*, *petD*, *rpl2*, *rpl16*, *rpoC1*, *trnA-UGC*, *trnG-UCC*, *trnI-GAU*, *trnK-UUU*, *trnL-UAA*, and *trnV-UAC*), and two two-intron genes (*rps12* and *ycf3*). Overall GC content of the whole genome was 33.9%.

To infer the phylogenetic relationships among *C. dielsiana* and its related taxa, whole cp genomes of 13 Papilionoideae species were downloaded from GenBank, which were aligned with that of *C. dielsiana* by applying MAFFT v.7 (Katoh and Standley 2013). We constructed a maximum-likelihood (ML) tree taking TIM2 as model, based on the alignment using IQ-TREE v.1.6. The result (Figure 1) showed that *Callerya*, *Sarcodum*, *Wisteria* were members of the monophyletic tribe Wisterieae (as in Compton et al. 2019; Duan et al. 2021). Among them, *Callerya dielsiana* and *C. nitida* were sister to *Wisteria*, and together formed a monophytic clade with *Sarcodum*. Wisterieae was nested in the inverted repeat-lacking clade (IRLC), which in turn belonged to the Hologalegina group of the subfamily Papilionoideae (see Wojciechowski et al. 2004; Schrire 2005).

**Figure 1.**
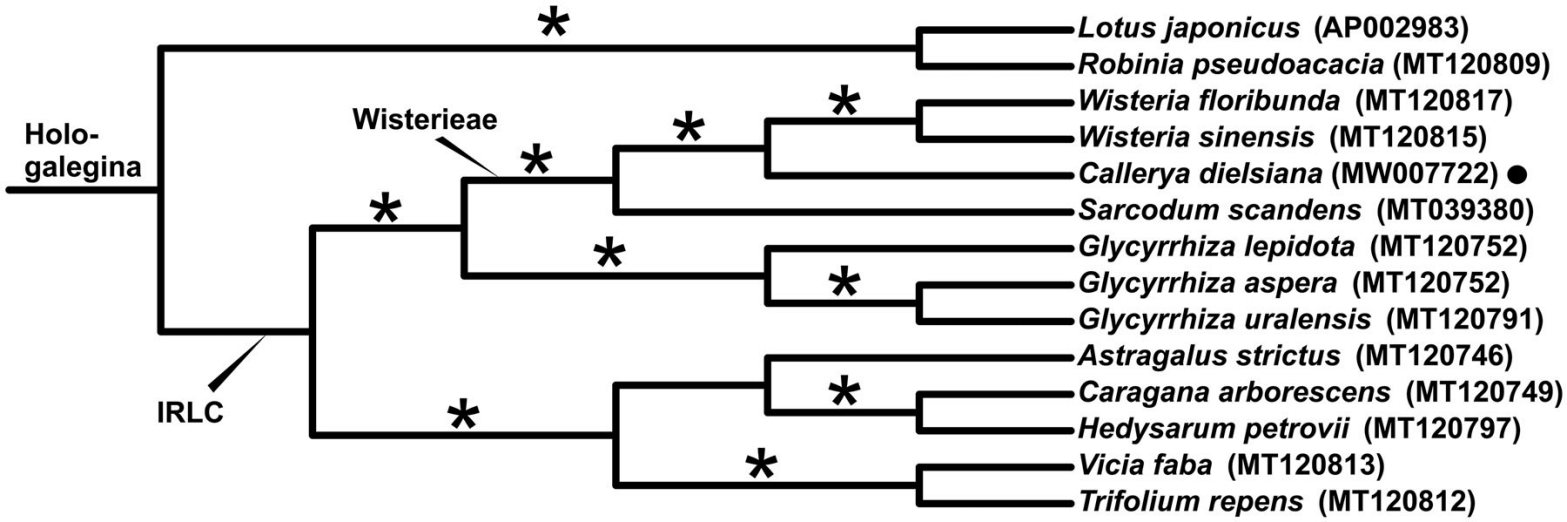
Maximum-likelihood (ML) phylogenetic tree based on 14 chloroplast genomes of Fabaceae. The position of *Callerya dielsiana* is indicated with black dot. The bootstrap values of 100% are shown on branches with asterisks.

## Ethical approval

This study includes no human, animal, or endangered plant samples, and the sample was legally collected in accordance with guidelines provided by the authors’ institution and national or international regulations. Field studies were complied with local legislation. No ethical approval/permission is required in this study.

## Author contributions

Qi-Fei Yi and Hong-Feng Chen involved in the conception, design and financial support; Ming-Chen Lin and Lin Fu collected the sample; Yong Tan and Lei Duan analyzed the data; Lei Duan drafted the paper, Li-Na Han revised the manuscript, and Qi-Fei Yi final approved the version to be published. All authors agree to be accountable for all aspects of the work.

## Data Availability

The genome sequence data that support the findings of this study are openly available in GenBank of NCBI at [https://www.ncbi.nlm.nih.gov] (https://www.ncbi.nlm.nih.gov/) under the accession no. MW007722. The associated BioProject, SRA, and Bio-Sample numbers are PRJNA706880, SRR13871830, and SAMN18131232, respectively.
